# Biological Misinterpretation of Transcriptional Signatures in Tumor Samples Can Unknowingly Undermine Mechanistic Understanding and Faithful Alignment with Preclinical Data

**DOI:** 10.1158/1078-0432.CCR-22-1102

**Published:** 2022-07-06

**Authors:** Natalie C. Fisher, Ryan M. Byrne, Holly Leslie, Colin Wood, Assya Legrini, Andrew J. Cameron, Baharak Ahmaderaghi, Shania M. Corry, Sudhir B. Malla, Raheleh Amirkhah, Aoife J. McCooey, Emily Rogan, Keara L. Redmond, Svetlana Sakhnevych, Enric Domingo, James Jackson, Maurice B. Loughrey, Simon Leedham, Tim Maughan, Mark Lawler, Owen J. Sansom, Felicity Lamrock, Viktor H. Koelzer, Nigel B. Jamieson, Philip D. Dunne

**Affiliations:** 1 The Patrick G Johnston Centre for Cancer Research, Queen's University Belfast, Belfast, United Kingdom.; 2 Wolfson Wohl Cancer Research Centre, Institute of Cancer Sciences, University of Glasgow, Glasgow, United Kingdom.; 3 School of Electronics, Electrical Engineering and Computer Science, Queen's University Belfast, Belfast, United Kingdom.; 4 University of Oxford, Oxford, United Kingdom.; 5 Information Services, Queen's University Belfast, Belfast, United Kingdom.; 6 Department of Cellular Pathology, Belfast Health and Social Care Trust, Belfast, United Kingdom.; 7 Cancer Research UK Beatson Institute, Glasgow, United Kingdom.; 8 Institute of Cancer Sciences, University of Glasgow, Glasgow, United Kingdom.; 9 School of Mathematics and Physics, Queen's University Belfast, Belfast, United Kingdom.; 10 Department of Pathology and Molecular Pathology, University and University Hospital of Zürich, Zürich, Switzerland.

## Abstract

**Purpose:**

Precise mechanism-based gene expression signatures (GES) have been developed in appropriate *in vitro* and *in vivo* model systems, to identify important cancer-related signaling processes. However, some GESs originally developed to represent specific disease processes, primarily with an epithelial cell focus, are being applied to heterogeneous tumor samples where the expression of the genes in the signature may no longer be epithelial-specific. Therefore, unknowingly, even small changes in tumor stroma percentage can directly influence GESs, undermining the intended mechanistic signaling.

**Experimental Design:**

Using colorectal cancer as an exemplar, we deployed numerous orthogonal profiling methodologies, including laser capture microdissection, flow cytometry, bulk and multiregional biopsy clinical samples, single-cell RNA sequencing and finally spatial transcriptomics, to perform a comprehensive assessment of the potential for the most widely used GESs to be influenced, or confounded, by stromal content in tumor tissue. To complement this work, we generated a freely-available resource, ConfoundR; https://confoundr.qub.ac.uk/, that enables users to test the extent of stromal influence on an unlimited number of the genes/signatures simultaneously across colorectal, breast, pancreatic, ovarian and prostate cancer datasets.

**Results:**

Findings presented here demonstrate the clear potential for misinterpretation of the meaning of GESs, due to widespread stromal influences, which in-turn can undermine faithful alignment between clinical samples and preclinical data/models, particularly cell lines and organoids, or tumor models not fully recapitulating the stromal and immune microenvironment.

**Conclusions:**

Efforts to faithfully align preclinical models of disease using phenotypically-designed GESs must ensure that the signatures themselves remain representative of the same biology when applied to clinical samples.

**Translational Relevance**

Integrity and robustness in aligning models with human tumors, based on comparable mechanistic and biological signaling representative of specific patient subtypes, is critical to ensure successful translation of preclinical understanding of disease and treatment efficacies into clinical benefit. The versatility and accessibility of transcriptional signatures renders them a fundamental tool in aligning clinical phenotypes and mechanisms across human tumors and preclinical models. Therefore, it is crucial to ensure that the biological meaning of transcriptional signatures remains faithful across different contexts.

In this study, we objectively measure the presence and extent to which the biology represented by transcriptional signatures can be altered by differences in tumor microenvironment conditions when moving between the preclinical and clinical settings. Importantly, we provide a freely-available resource (https://confoundr.qub.ac.uk) that allows users to test the extent to which their gene or signatures of interest might become confounded due to the stromal transcriptome during forward and reverse translation.

## Introduction

Although the publication of gene expression-based signatures (GES) continues to grow each year in the research setting, these published signatures rarely make any clinical impact (1). In addition to potentially-addressable technical confounders, such as sample size issues or lack of validation cohorts, the biology underpinning the signature may also expose a critical weakness in current translational bioinformatics research pipelines, when applied to clinical samples either in retrospective collections or prospective trials. This is particularly pertinent as researchers now have unparalleled access to cancer datasets that can be routinely characterized using the tens of thousands of GESs already available in molecular databases, such as The Cancer Genome Atlas (TCGA) (2). The contribution of the stroma to the cancer transcriptome is well established and has been the subject of numerous studies (3, 4). We and others have previously demonstrated the confounding effects of stroma on molecular subtypes in colorectal cancer (5, 6), alongside specific influences of the tumor microenvironment on epithelial–mesenchymal transition (EMT)-related signatures (7, 8). While such studies have clearly defined the importance of the stroma, there remains a need for a more detailed assessment, and subsequent enumeration, of the consequences of the stromal influence on gene expression in terms of pathway and ontology associations and subsequent biological interpretation of the resulting GESs.

A number of recent studies have highlighted the characterization required to ensure faithful alignment between human tumors and preclinical models, in terms of the biological signaling and therapeutic responses in both (9–11). Integrity and robustness in aligning models with human tumors is critical in the era of precision medicine, where treatments are tailored for the biology underpinning specific cancer subtypes. Furthermore, signature development and testing is increasingly performed in disease-matched preclinical models, using *in vitro*, *in vivo*, or *ex vivo* systems, enabling almost absolute control over the experimental conditions employed during biology-driven GES development (12–14). Although such “clean” models are exquisitely suited for precise identification and characterization of discrete mechanistic signaling, when compared with the relative unpredictable nature of diagnostic sample acquisition, differences in the epithelial, immune, and stromal composition between the models and clinical samples (15) has the potential to confound our understanding and interpretation of these signatures in specific domains. While this will in no way alter the prognostic/predictive statistical value of such signatures, differences in cellular composition and tumor stroma percentage (TSP) might not be accounted for during the interpretation of the true biological meaning of the GES result in bulk tumor datasets. Conversely, when biomarkers of prognosis, response or molecular subtypes are identified from tumor datasets, approaches to reverse-translate these findings into preclinical models introduces the potential for assessment of these genes/signatures in lineages that do not represent the true cellular source of the signaling in clinical samples.

The prognostic value of stromal content in cancer can be reproduced using molecular or histologic methods, and is one of the most robust predictors of relapse in stage II/III colorectal cancer (CRC), where tumors with a higher tumor stroma percentage (TSP) are associated with poor outcomes (6, 16–19). It is important to note that the statistical correlation between a specific biomarker/signature and a clinical variable like relapse, are in no way weakened if the end-user does not accurately consider the true biological interpretation of the signature itself. As such, for GESs that aim to provide the most statistically significant prognostic/predictive value, the true meaning of the biology underpinning the signature may be irrelevant, however, for GESs that are designed to represent mechanistic biology, the integrity of the biology they represent is critical. While biological researchers understand that correlation does not always equate to causation (20), there remains a potential gap in our understanding when interpretation of GESs can be influenced by the cellular composition of a tumor sample. The potential for misinterpretation is an issue that has become even more important in the precision medicine era (21), where it is now essential that therapeutic targeting is based on robust and accurate mechanistic-driven evidence performed in models that are representative of specific patient subtypes (9).

To examine if variations in TSP can distort GES results, which in turn could lead to biological misinterpretation, we performed a comprehensive assessment and quantification of the extent that stromal composition in bulk tumors can skew the expression levels of *n* = 7,835 of commonly employed gene sets and signatures in cancer research. Using a combination of discovery and independent validation cohorts, including tissues from laser capture microdissection (LCM), flow cytometry, bulk clinical samples, single-cell RNA sequencing (scRNA-seq) and finally spatial transcriptomics, enabled a detailed interrogation of widely used transcriptomic signatures to enumerate the extent to which stromal composition can confound their classification. The pan-cancer nature of these findings were subsequently assessed across a number of publicly-available LCM datasets derived from pancreatic, breast, ovarian, and prostate cancer. Furthermore, to ensure that our findings can be widely applied, we have developed the freely-available ConfoundR online resource; https://confoundr.qub.ac.uk/, which gives a user the ability to quickly and easily interrogate the potential confounding effects on any individual gene, combination of genes, and GES across colorectal, pancreatic, breast, ovarian, and prostate cancer datasets.

## Materials and Methods

### Datasets

When publicly available, the data were assessed via Gene Expression Omnibus (GEO) and the processed data matrix downloaded. All array data were collapsed using the collapseRows function within weighted gene co-expression network analysis (WGCNA) (RRID:SCR_003302; v1.70–3) R package. In the case of duplicated genes, the probe with the highest mean expression across all samples was used and those genes with no expression across the dataset were removed. All GEO datasets are available via https://www.ncbi.nlm.nih.gov/geo/ using gene series (GSE) codes below:

*Discovery: LCM* GSE35602; matched epithelium and stroma from 13 colorectal tumors transcriptionally profiled using Aligent array. *Validation: LCM* GSE31279; matched epithelium and stroma from eight colorectal tumors (with both compartments) transcriptionally profiled using Illumina sentrix-8 chip. *Validation: FACS* GSE39396; Six CRC tumors were sorted by Fluorescence-activated cell sorting (FACS) into four cell populations: epithelial cells (EPCAM^+^), leukocytes (CD45^+^), fibroblasts [fibroblast activated protein (FAP^+^)] and endothelial cells (CD31^+^). *Validation: Breast Cancer* GSE14548; matched LCM epithelium and stroma from nine invasive ductal carcinomas transcriptionally profiled using the Affymetrix Human X3P Array. *Validation: TNBC* GSE81838; matched LCM epithelium and stroma from 10 triple-negative breast cancers (TNBC) transcriptionally profiled using the Affymetrix Human Gene 1.0 ST Array. *Validation: PDAC* GSE164665; matched LCM epithelium and stroma from 19 pancreatic ductal adenocarcinomas (PDAC) transcriptionally profiled by Illumina NextSeq 500. *Validation: Ovarian Cancer* GSE9899; matched LCM epithelium and stroma from five ovarian tumors transcriptionally profiled using the Affymetrix Human Genome U133 Plus 2.0 Array. *Validation: Prostate Cancer* GSE97284; matched LCM epithelium and stroma from 25 prostate tumors, of which 12 were low-grade (Gleason 3 + 3) and 13 were high-grade (Gleason 8 or above) transcriptionally profiled using the Affymetrix Human Gene 1.0 ST Array. *Clinical Validation: FOCUS trial* GSE156915; The UK Medical Research Council FOCUS [Fluorouracil, Oxaliplatin and CPT11 (irinotecan)] trial involving patients with stage IV primary CRC resection transcriptionally profiled on Almac Xcell chip, only those with matched histology remained for analysis (*n* = 356). *Validation: scRNA-Seq* GSE144735; from 6 colorectal patients were single-cell sequenced. Count matrix was aligned to annotation file within Partek Genomics Suite. Genes with an expression less than 1 in at least 99.9% of cells were removed. Data was normalized by counts per million, +1 and log_2_ transformed. *Validation: BOSS biopsy* GSE85043; multiple biopsies obtained with different regions of seven CRC resection specimens, profiled on Affymetrix array.

#### GeoMx digital spatial profiler

The whole slide was imaged at 20× magnification using the GeoMx digital spatial profiler (DSP; RRID:SCR_021660) with the integrated software suite then used to select 300 to 600 μM diameter regions of interest (ROI) from which the instrument focuses UV light (385 nm), to cleave the UV-sensitive probes with the subsequent release of the hydridized barcodes. 11 ROIs corresponding to epithelial tumor center, abundant tumor microenvironment (TME) regions and regions representing an interface between tumor and TME and were selected. The DSP software enabled Areas of Interest (AOI) contained in individual ROIs to be defined and selected. Firstly segments containing Pan- Cytokeratin (PanCK^+^) immunofluorescence (IF) signal were masked for tumor epithelium and extracted, then the complementary inverse segments (PanCK^−^) was masked and captured corresponding to the TME. For additional information see Supplementary Methods.

### Digital histology scoring

Hematoxylin and eosins (H&E) from FOCUS (*N* = 356) were scanned at high resolution on an Aperio scanner at a total magnification of 20x. Tissue segmentation was run on H&E images by deep convoluted neural net (Very Deep Convolutional Networks for Large-Scale Image Recognition; ref. 22) using the HALO platform (RRID:SCR_018350; Indica Labs). Supervised training had been performed using more than 1,500 tissue areas, combining visual pathologic review and deep convoluted neural network, from four CRC cohorts as previously described (23). Counts of single cells were utilized to assess the proportion of desmoplastic stroma (DS) compared with total cell counts.

### Data analysis

***MCP.*** MCPcounter (v1.2.0) R package was used to generate scores for 10 cell populations. ***Consensus Molecular Subtype.*** Consensus Molecular Subtype (CMS) classification utilized “classifyCMS.SSP” function within the CMSclassifier (v1.0.0) R package and the CMScaller (v2.0.1) R package. ***Single-sample gene set enrichment analysis.*** Single-sample gene set enrichment analysis (ssGSEA) was performed using gsva (RRID:SCR_021058; v1.38.2) R package with the following nondefault settings: min.sz = 5, verbose = TRUE, method = “ssgsea”, on the HALLMARK, Gene Ontology: Biological Processes and the Kyoto Encyclopedia of Genes and Genomes (KEGG) gene sets. ESCAPE (v1.4) R package was utilized to generated single sample scores for the HALLMARK pathways within the single cell cohort using the “enrichIt” function: groups = 1,000, cores = 2. ***GSEA.*** Pair-wise GSEA was performed using fgsea (RRID:SCR_020938; v1.16.0) R package (minSize = 1, maxSize = Inf, nperm = 10,000), on the HALLMARK, Gene Ontology: Biological processes and the KEGG gene sets accessed via msigdb (RRID:SCR_016863; v7.4.1). Within the FOCUS validation dataset a median split of DS was used for comparison, followed by Differential gene set enrichment analysis (DGEA). ***DoRothEA.*** Transcriptional factor activity was assessed using dorothea (v1.2.2) R package, within the “run_viper” function (filtered for high confidence regulons). Within LCM cohorts, rowTtest with a *P* < 0.05 was considered significant to obtain consensus LCM transcription factors (TF). Plots in subsequent cohorts include all TF, regardless of significance. **ESTIMATE.** estimate (v1.0.13) R package was used to generate stromal and immune scores. R studio (v1.3.1073), R Project for Statistical Computing (RRID:SCR_001905; v4.0) used for all analysis. All heatmaps were plotted using ComplexHeatmap (RRID:SCR_017270) and all additional plots using ggplot2 (RRID:SCR_014601).

### ConfoundR Shiny application

#### Development and access

The ConfoundR application was created using R version 4.1.2 in combination with the R package shiny (RRID:SCR_001626; v1.7.1) and is running on the Shiny Server (v1.5.17) hosted on the Queen's University Belfast (Belfast, UK) virtual server (CentOS 7, 64-bit, Intel Xeon Gold E5–2660 v3 @2.60 GHZ, 16 Core). ConfoundR is accessible at https://confoundr.qub.ac.uk. Datasets: The datasets used in the ConfoundR application are described above, along with the preprocessing methods applied to each dataset.

#### Expression boxplots

The Expression Boxplots module allows the user to enter the gene symbol for a single gene into the input box. Boxplots in the Expression Boxplots module are created using ggplot2 (RRID:SCR_014601; v3.3.5) and Mann–Whitney *U* tests are performed using the stat_compare_means function from the ggpubr package (RRID:SCR_021139; v0.4.0) with method = “wilcox.test”. Boxplots for the PDAC dataset (GSE164665) are plotted using normalized counts calculated by DESeq2 (RRID:SCR_015687; v1.34.0), using the size factors calculated by the estimateSizeFactors function, accessed via the counts function with normalized = TRUE. Plots for each of the datasets can be downloaded in png format using the Save Plot button.

#### Expression Heatmap

The Expression Heatmap module enables users to enter a list of gene symbols with each gene symbol on a new line. For the RNA-seq dataset, variance stabilizing transformed counts, calculated using the vst function (blind = FALSE), from the DESeq2 package (RRID:SCR_015687; v1.34.0), are used as the gene expression values for samples. The gene expression values for each user selected gene in each dataset are converted to *Z*-scores using the scale function (center = TRUE, scale = TRUE) prior to plotting heatmaps. Heatmaps of the gene expression *Z*-scores are plotted using the ComplexHeatmap package (RRID:SCR_017270; v2.10.0) with the samples grouped by the respective cell/tissue types to aid visual comparison between groups.

#### GSEA

The GSEA module enables users to select an existing gene set from established gene set collections using dropdown menus or to enter a custom user-defined gene set by entering a list of gene symbols with each symbol on a new line. The existing gene sets available to the user are the Hallmark, KEGG, Reactome, BioCarta, and Pathway Interactions Database (PID) gene sets as curated by the Molecular Signatures Database (MSigDB; RRID:SCR_016863) and accessed via the msigdbr package (v7.4.1).

In order to perform preranked GSEA, differential analysis is performed for each of the datasets, comparing stromal samples with epithelial samples. For the GSE39396 dataset the user can specify the cell types (epithelial, leukocytes, endothelial, fibroblasts) to compare using the input boxes provided. Differential analysis is performed using limma (RRID:SCR_010943; v3.50.0) for microarray datasets (GSE39396, GSE35602, GSE31279, GSE81838, GSE14548, GSE9899, GSE97284) and using DESeq2 (RRID:SCR_015687; v1.34.0) for the RNA-seq dataset (GSE164665). Following differential analysis, genes are ranked according to the *t*-statistic (limma) or Wald statistic (DESeq2). Preranked GSEA is performed by the GSEA function from the clusterProfiler package (RRID:SCR_016884, v4.2.1) using the fgseaSimple method with 10,000 permutations (by = “fgsea”, nPerm = 10,000) and a random seed of 123. Plots of GSEA results are produced using a modified version of the gseaplot2 function from the enrichplot package.

#### Packages used

The ConfoundR app uses the following R packages: shiny (v1.7.1), shinydashboard (v0.7.2), dashboardthemes (v1.1.5), shinyFeedback (v0.4.0), shinybusy (v0.2.2), shinyccssloaders (v1.0.0), msigdbr (v7.4.1), ggplot2 (v3.3.5), cowplot(v1.1.1), ggbeeswarm (v0.6.0), ggpubr (v0.4.0), ComplexHeatmap (v2.10.0), limma (v3.50.0), DESeq2 (v1.34.0), clusterProfiler (v4.2.1), fgsea (v1.20.0), enrichplot (v1.14.1) and RColorBrewer (v1.1–2). Schematics drawn using BioRender.

#### Data availability statement

The data analyzed in this study were obtained from GEO at GSE35602, GSE31279, GSE39396, GSE81838, GSE14548, GSE9899, GSE97284, GSE164665, GSE156915, GSE144735, and GSE85043. The source code for the ConfoundR app is available at https://www.github.com/Dunne-Group/ConfoundR. All scripts to perform the analyses outlined in this paper are available on our lab website www.Dunne-Lab.com.

## Results

### Initial characterization of tumor epithelium and stromal datasets

**Figure 1.**
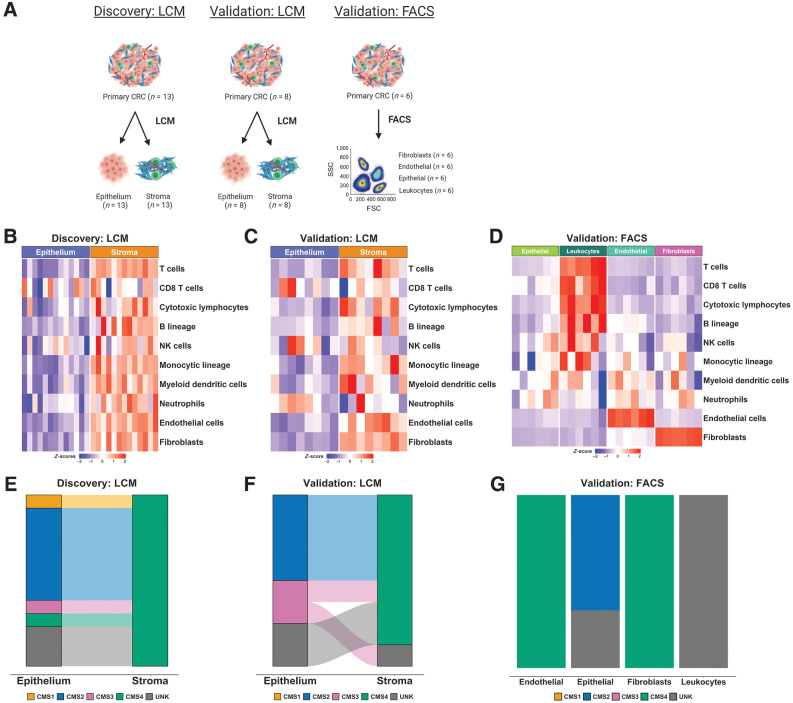
Initial characterization of tumor epithelium and stromal datasets. **A,** Schematic of the segregation strategies in the discovery and validation cohorts, drawn using BioRender. **B,** Heatmap of MCP-counter scores for the LCM discovery cohort, according to epithelium and stromal regions. **C,** Heatmap of MCP-counter scores for the LCM validation cohort, according to epithelium and stromal regions. **D,** Heatmap of MCP-counter scores for the FACS validation cohort. **E,** CMS classifications (using CMSclassifier) for the matched epithelium and stroma samples in the LCM discovery cohort. **F,** CMS calls (using CMSclassifier) for the matched epithelium and stroma samples in the laser capture microdissected validation cohort. **G,** CMS calls (using CMSclassifier) for the four lineages in the FACS validation cohort. CMS, consensus molecular subtypes; FSC, forward light scatter; LCM, laser capture microdissection; MCP, microenvironment cell population; SSC, side light scatter.

To assess the influence that TSP has on commonly used transcriptional signatures, we designed a study to identify, characterize, and orthogonally validate the TME compartments and lineages associated with specific transcriptional signatures within primary CRC. This approach utilized a series of independent primary tumor samples that had undergone LCM, to segregate tissue into epithelial and stromal components, for discovery (*n* = 26 samples from *n* = 13 tumors; GSE35602) and validation (*n* = 16 samples from *n* = 8 tumors; GSE31279; Fig. 1A). Further delineation of cell-type–specific transcriptional signaling was performed using transcriptional data generated from FACS-purified epithelial, fibroblast, endothelial, and leukocyte cell populations from colorectal cancer resections (*n* = 6 tumors, *n* = 24 populations; GSE39396; Fig. 1A).

To confirm the purity of these datasets, we utilized the microenvironment cell population (MCP)-counter algorithm (24) to assign single sample scores for *n* = 10 stromal [fibroblasts, endothelial cells) and immune lineages (T cells, CD8 T cells, cytotoxic lymphocytes, B lineages, natural killer (NK) cells, monocytic lineages, myeloid dendritic cells, neutrophils] to each individual sample (Fig. 1B–D). In the LCM cohorts, these analyses confirmed that the majority of TME lineage signatures are exclusively stromal, particularly those aligned to fibroblast and endothelial cells (Fig. 1B and C). Although most immune lineages seemed to align to the stroma, we did observe signaling indicative of CD8 T cells, NK cells, and neutrophils within the epithelial compartment, indicative of intraepithelial infiltration of these specific immune lineages (Fig. 1B and C). In line with this LCM data, within the FACS cohort we observed fibroblast and endothelial populations aligned exclusively to the MCP-counter signature for fibroblasts and endothelial cells respectively, supporting the suitability of our approach and the utility of the MCP-counter signatures (Fig. 1D). While T-cell, CD8 T-cell, cytotoxic lymphocyte, and B-cell lineage scores all closely aligned to the purified leukocyte population as expected, we did observe signaling indicative of NK cells, myeloid dendritic cells, and neutrophils in non-leukocyte populations, suggesting that there was some crossover in these specific populations during cell sorting for epithelial cells (EPCAM^+^), leukocytes (CD45^+^), fibroblasts (FAP^+^), and endothelial cells (CD31^+^), or that the signatures cannot be used for precise enumeration of these lineages in CRC tissue.

### Association of colorectal cancer molecular subtypes with stromal components

A number of studies including our own have identified the stromal and immune contributions to the CRC CMS (3, 5, 6, 19), in particular to CMS1 and CMS4, however the relative contributions of TME compartments and specific lineage contributing to CMS calls using the original classifier have not been detailed. To test this, we classified the epithelial and stromal components from each tumor using the CMSclassifier (19) algorithm, where we found that with the exception of one unclassified sample (UNK), the stroma was exclusively classified as CMS4 in the LCM cohorts (Fig. 1E and F), as were both the purified fibroblast and endothelial lineages in the FACS cohort (Fig. 1G), suggesting that transcriptional signaling from these components alone, even in the absence of the epithelial transcriptome, is sufficient for tumor classification as CMS4, the group with the worst prognosis in CRC.

When the epithelium was examined, with the exception of two samples, we observed a strong tendency for classification of CMS2 and CMS3, both well-characterized epithelial-rich subtypes, across the LCM and FACS cohorts (Fig. 1E–G). In contrast to the association between stromal/endothelial cells and CMS4, when the leukocyte FACS purified population calls were assessed, we observed uniform unknown/unclassified assignments, indicating that the presence of immune infiltration alone is not sufficient for classification of a tumor as an immune-rich CMS1 tumor (Fig. 1G) and more complex histologic features involving tumor infiltrating lymphocytes and epithelial components are required. Furthermore, these issues remain apparent when using the CMScaller classifier (25), specifically modified to classify epithelial-based preclinical models according to CMS (Supplementary Fig. S1).

### Stromal influence on widely used transcriptional signatures

**Figure 2.**
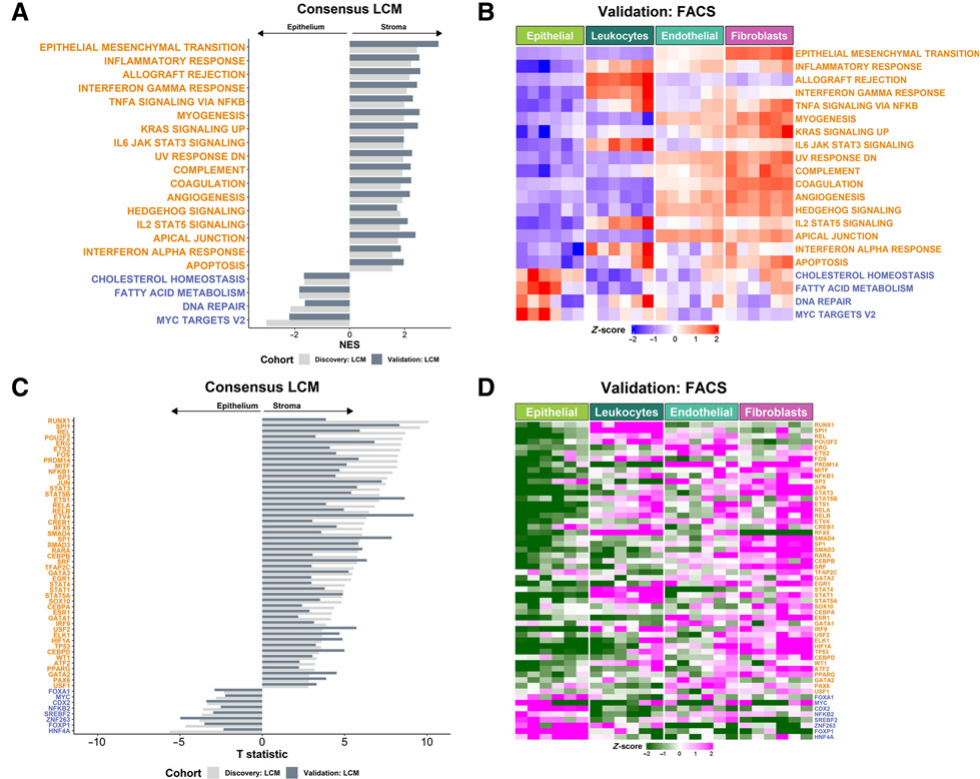
Stromal influence on widely used transcriptional signatures. **A,** GSEA of Hallmark gene sets in LCM discovery and validation cohorts. Only gene sets significantly and concordantly enriched in stroma or epithelium in both the discovery and validation cohorts are shown (*P*_adj_ < 0.02). **B,** Heatmap of ssGSEA scores for the Hallmark gene sets in the FACS validation cohort samples. Only the gene sets significantly and concordantly enriched in stroma or epithelium in both the LCM discovery and validation cohorts are shown (*P*_adj_ < 0.02). **C,** TFs whose activity was significantly and concordantly enriched in stroma or epithelium in both the LCM discovery and validation cohorts (*P* < 0.05). **D,** Heatmap of the inferred activity scores for the same TFs in the FACS validation cohort. For all panels in Fig. 2, gene sets/transcription factors with names/symbols colored orange were significantly and consistently enriched in stroma in the LCM discovery and validation cohorts, whereas gene sets/TFs with names/symbols colored blue were consistently and significantly enriched in epithelium in the LCM discovery and validation cohorts (gene sets: *P*_adj_ < 0.02; TFs: *P* < 0.05). LCM, laser capture microdissection; NES, normalized enrichment scale; TF, transcription factor.

While individual studies have highlighted the stromal origins of a number of key genes/proteins, using methods similar to MCP, it remains unknown how influential the stromal transcriptome is on some of the most widely employed GESs. To investigate this, we performed pair-wise GSEA (26) comparing epithelium to stroma using one of the most commonly used pathway/ontology collections, the MSigDB (27) of *n* = 50 “Hallmarks” (Supplementary Fig. S2A and S2B). By performing these analyses in both LCM cohorts in tandem, we observed that *n* = 21 Hallmarks were significantly [*P*_adjusted_ (*P*_adj_) < 0.02; more stringent that the accepted 0.25 cut-off] and consistently associated with either stroma (*n* = 17) or epithelium (*n* = 4) across both LCM cohorts (Fig. 2A). These findings were further validated using ssGSEA in the FACS cohort (Supplementary Fig. S2C), where the *n* = 17 stromal-associated and *n* = 4 epithelial-associated Hallmarks were again enriched in the corresponding cell populations (Fig. 2B). Despite being developed and named to classify samples associated with specific biology, these analyses reveal the signaling underpinning these signatures may be entirely, albeit unintentionally, misinterpreted due to the confounding effects of the stromal transcriptome in bulk tumor data. To ensure that this confounding effect is not an artifact of the Hallmark signatures specifically, we performed the same analyses using the *n* = 186 KEGG and *n* = 7,481 gene ontology biological processes (GO BP) signatures, where again we found widespread stromal influence in 50 of 186 (Supplementary Fig. S2D) and 949 of 7,481 (Supplementary Table S1) signatures consistently in both cohorts, validated within the FACS cohort (Supplementary Fig. S2E and S2F).

We also observed similar confounding effects at the TF activity level, when assessed using the *n* = 118 defined regulons within the Dorothea algorithm.(28) These analyses revealed the extent to which numerous seemingly epithelial-specific cancer-associated TFs are influenced by stromal content, across both LCM cohorts (Fig. 2C); with *n* = 48 TFs significantly activated in stromal components, compared with only *n* = 8 TFs being significantly activated in the epithelium. As with the transcriptional signatures, when extended into the FACS purified populations, we observed a near identical overlap with the LCM findings and identified a number of lineage-specific associations (Fig. 2D). Given the potential implications of the CRC findings described above, we next questioned if this was a pan-cancer phenomena, by performing the same analysis in LCM cohorts from breast cancer, TNBC, PDAC, ovarian cancer, and prostate cancer. Despite some small organ-specific discrepancies in individual GESs, these analyses again revealed that the presence and extent of the confounding effect of the stroma is not CRC–specific, highlighting the potential for widespread biological misinterpretation of these signaling pathways across multiple cancer types (Supplementary Fig. S2G–S2K).

### The ConfoundR resource enables estimation of stromal influence on transcriptional signatures simultaneously across multiple cancer types

**Figure 3.**
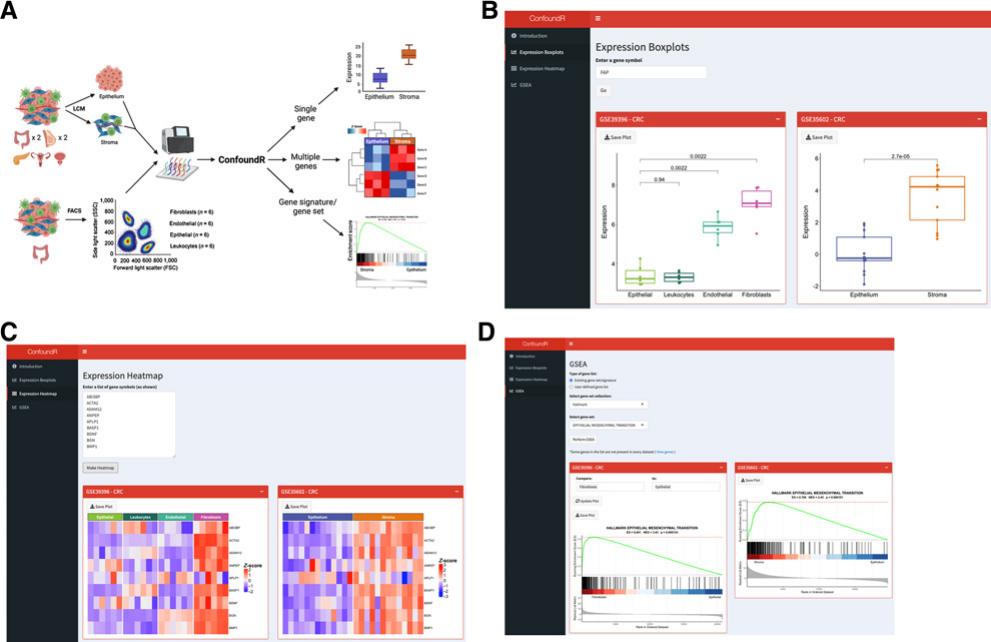
The ConfoundR resource enables stromal influence estimation in cancer tissue. **A,** Schematic overview of the cohorts and analyses available within the ConfoundR app, accessible via https://confoundr.qub.ac.uk/. **B,** Expression Boxplots analysis module of ConfoundR enabling the expression of a single gene to be compared between stroma and epithelium samples in each of the ConfoundR datasets. **C,** Expression Heatmap analysis module of ConfoundR enabling the expression of multiple genes to be visually compared between stroma and epithelium samples in each of the ConfoundR datasets. **D,** GSEA analysis module of ConfoundR allowing GSEA of existing gene sets from established gene set collections or custom user defined gene sets to be performed comparing stroma with epithelium in each of the ConfoundR datasets.

Our findings of the presence of the stromal confounding effect across cancers, coupled with the widespread interest in biomarker/GES identification and application, motivated us to develop the online resource, ConfoundR (https://confoundr.qub.ac.uk/). ConfoundR enables users, regardless of their bioinformatics skillset, to examine individual genes, combinations of genes, and GES of interest to identify if they could be susceptible to the same stromal confounding issues we have identified in this study. This freely available online resource enables users to interrogate the CRC, breast cancer, TNBC, PDAC, ovarian cancer, and prostate cancer datasets through three analysis modules: gene expression boxplots, gene expression heatmaps, and GSEA (Fig. 3A).

The “Expression Boxplots” module of ConfoundR allows gene expression comparisons of a single gene between epithelial samples and stroma samples in each dataset, by creating boxplots and providing accompanying *P* values for Mann–Whitney *U* tests (Fig. 3B). ConfoundR's “Expression Heatmap” module allows expression levels of multiple genes to be visually compared between epithelial and stromal samples in each dataset using a heatmap (Fig. 3C). Finally, the GSEA module of ConfoundR, enables the user to perform GSEA comparing stromal and epithelial samples in each dataset from established gene set collections: Hallmarks (*n* = 50), KEGG (*n* = 186), Reactome (*n* = 1,604), BioCarta (*n* = 292), and PID (*n* = 196). In addition, as many researchers will be interested in assessing their own bespoke or unpublished gene signatures, ConfoundR also provides the end-user with complete control to input and generate GSEA results from an unlimited number of custom gene sets (Fig. 3D). To exemplify the utility of the ConfoundR resource, we examined the expression of the *FAP* gene using the Expression Boxplots module, the expression of a subset of genes from the Hallmark EMT gene set using the Expression Heatmap module and the GSEA module to perform GSEA for the Hallmark EMT gene set (Fig. 3B-D). The ConfoundR application provides all cancer researchers with a freely available and novel resource to test the susceptibility of any gene, lists of genes, or gene signatures to the stromal confounding phenomenon described in this study.

### Application of findings to bulk colorectal cancer tumor data

**Figure 4.**
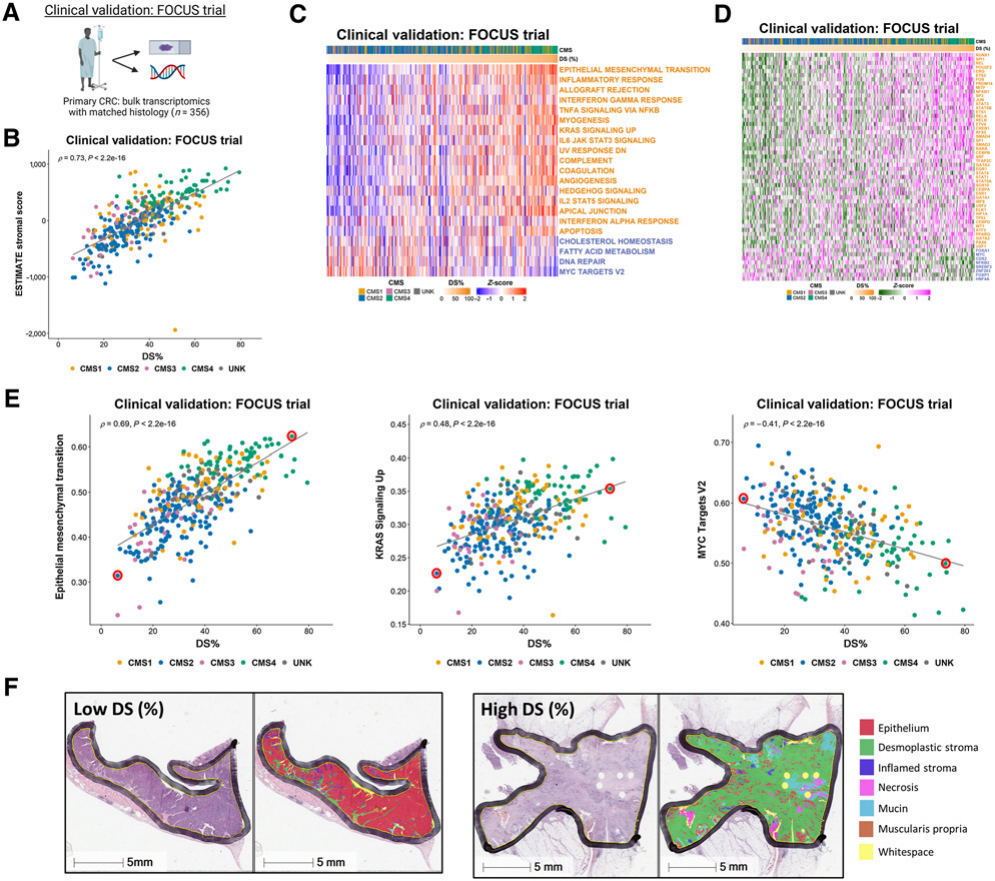
Application of findings to bulk colorectal cancer tumor data. **A,** Schematic summary of the clinical validation dataset from the FOCUS clinical trial. **B,** Scatterplot showing correlation between desmoplastic stroma percentage (DS%) determined from H&E assessment and ESTIMATE Stromal Score determined by transcriptomic data in the FOCUS clinical trial samples (Spearman rho = 0.73, *P* < 2.2e-16), colored by CMS calls (CMS1: *n* = 62; CMS2: *n* = 155; CMS3: *n* = 29; CMS4: *n* = 66; UNK: *n* = 44). **C,** Heatmap of ssGSEA scores for the Hallmark gene sets (identified in Fig. 2 as significantly enriched in the stroma/epithelium in the LCM discovery and validation cohorts) for the FOCUS clinical trial samples. Samples ranked in order of DS% from lowest (left) to highest (right). Gene sets with names colored orange were significantly enriched in stroma in the LCM discovery and LCM validation cohorts and gene sets with names colored blue were significantly enriched in epithelium in the LCM discovery and LCM validation cohorts. **D,** Heatmap of activity scores for TFs (identified as significantly enriched in the stroma/epithelium in the LCM discovery and validation cohorts) for the FOCUS clinical trial samples. Samples are arranged in order of DS% from lowest (left) to highest (right). Gene sets with names colored orange were significantly enriched in stroma in the LCM discovery and LCM validation cohorts and gene sets with names colored blue were significantly enriched in epithelium in the LCM discovery and LCM validation cohorts. **E,** Scatterplots showing the correlation between DS% determined from H&E and ssGSEA scores for the Epithelial Mesenchymal Transition (left; Spearman rho = 0.69, *P* < 2.2e-16), KRAS Signaling Up (middle; Spearman rho = 0.48, *P* < 2.2e-16) and MYC Targets V2 (right; Spearman rho = -0.41, *P* < 2.2e-16) Hallmark gene sets. We identified two cases representative of low and high DS% in each of these analyses (red circles). **F,** H&E along with HALO mark-up for the representative low and high DS% samples identified in (**E**). CRC, colorectal cancer; LCM, laser capture microdissection.

To test these findings further in bulk tumor datasets, we utilized transcriptional data from *n* = 356 primary tumors used in the FOCUS clinical trial (Fig. 4A; GSE156915; ref. 29), alongside digital pathology-derived DS percentage (DS%) score derived from H&Es (detailed in Methods). We confirmed the previously-reported associations between CMS4 and stromal content are also observed in this tumor cohort (Supplementary Fig. S3A) alongside strong correlation between our digital pathology assessments of stroma and the MCP fibroblast score (ρ = 0.64, *P* < 2.2e-16; Supplementary Fig. S3B) and ESTIMATE (30) stromal score (ρ = 0.73, *P* < 2.2e-16; Fig. 4B). Using DS% to rank the tumor samples from low to high, we next assessed all the Hallmarks (Supplementary Fig. S3C), alongside the subset of Hallmarks and TFs that were found to be significantly associated with stroma/epithelium in the LCM and FACS cohorts (Fig. 4C and D), revealing a strikingly clear pattern that again indicates how widely the stromal components of a tumor can confound the interpretation of transcriptional signatures and TF activity scores in the bulk tumor setting. The Hallmarks associated with the immune component within the FACS validation analysis (*n* = 7; Supplementary Fig. S2C), ranked by DS content, also correlated to DS% (Supplementary Fig. S3D).

Throughout our analyses a number of individual signatures were consistently associated with the strongest confounding effects of stromal content, and therefore we selected these as specific exemplars related to DS%; namely the EPITHELIAL MESENCHYMAL TRANSITION (Spearman rho = 0.69, *P* < 2.2e-16), KRAS SIGNALING UP (Spearman rho = 0.48, *P* < 2.2e-16), and MYC TARGETS V2 (Spearman rho = -0.41, *P* < 2.2e-16; Fig. 4E) Hallmark signatures. We identified two cases representative of low and high DS% in each of these analyses (Fig. 4E, red circles) and assessed histologic features according to H&Es with Artificial intelligence (AI)-guided tissue segmentation, which provided a visual confirmation that these Hallmark signatures are confounded by quantity of DS across the tissue section (Fig. 4F).

### Lineage-specific scRNA-seq assessment of the Hallmarks EMT signature

**Figure 5.**
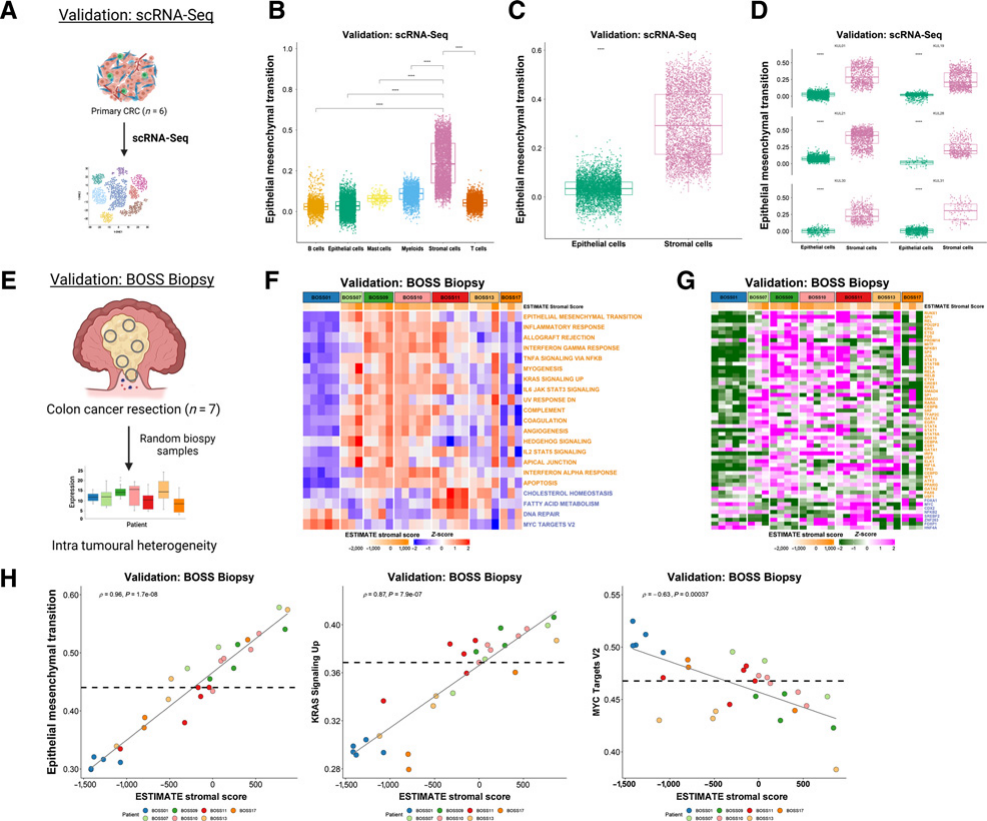
Single-cell and multi-regional biopsy analyses. **A,** Schematic of scRNA-seq cohort derived from *n* = 6 CRC primary tumors. Boxplots showing ssGSEA scores for the Hallmark Epithelial Mesenchymal Transition gene set across the various cell types (**B**) and specifically between epithelial and stromal cells (**C**; from all six colorectal cancer tumors) in the scRNA-seq dataset (*P* < 2.2 × 10–16; Wilcoxon test). **D,** Comparison of ssGSEA scores for the Hallmark Epithelial Mesenchymal Transition gene set between epithelial and stromal cells in each primary CRC (*n* = 6) in the scRNA-seq dataset (all *P* < 2.2×10–16; Wilcoxon test). Epithelial cells are shown in green and stromal cells in pink. **E,** Schematic overview of the BOSS Biopsy cohort consisting of colon cancer resections from 7 patients each with up to *n* = 5 multi-regional biopsy samples. Heatmaps of ssGSEA scores for the Hallmark gene sets (**F**) and TF activity scores for the BOSS Biopsy samples (**G**). Samples are grouped according to patient of origin and the ESTIMATE Stromal Score of each biopsy sample is indicated by the ESTIMATE StromalScore bar at the top of the heatmap. Only the gene sets/TFs significantly and concordantly enriched in stroma or epithelium in both the LCM discovery and LCM validation cohorts are shown (from Fig. 2; *P*_adj_ < 0.02 – Hallmarks; *P* < 0.05 – TFs). Gene sets/TFs with names/symbols colored orange were significantly enriched in stroma in the LCM discovery and LCM validation cohorts and gene sets/transcription factors with names/symbols colored blue were significantly enriched in epithelium in the LCM discovery and LCM validation cohorts. **H,** Scatterplots showing correlation between the ESTIMATE StromalScore and ssGSEA scores for the Hallmark Epithelial Mesenchymal Transition (left; Spearman rho = 0.96, *P* = 1.7e-08), KRAS Signaling Up (middle; Spearman rho = 0.87, *P* = 7.9e-07) and MYC Targets V2 (right; Spearman rho = −0.63, *P* = 0.00037) gene sets. Samples are colored by patient of origin. CRC, colorectal cancer.

scRNA-seq can be deployed to provide exceptional lineage-specific resolution in transcriptional studies, and this method has been used to great effect in the identification of tumor heterogeneity and phenotypic associations (31). To assess how far our findings extend in such data, we utilized a scRNA-seq cohort derived from *n* = 6 CRC primary tumors (Fig. 5A; GSE144735), where across all regions at a single-cell resolution the Hallmark EMT signature displays a significant enrichment in stromal cells compared with all other cell types (*P* < 2.2e-16; Fig. 5B) and in particular when comparing epithelial and stromal only (*P* < 2.2e-16; Fig. 5C). The highest EMT scoring epithelial cells only ever display an EMT gene expression signature score that reaches the lowest quartile of EMT signature score for stromal cells across all samples (Fig. 5D). Based on these data, despite EMT signatures proving to be highly-tractable biomarkers of epithelial cells undergoing transitions when utilized in *in vitro*, preclinical, or scRNA-seq data, these data provide further proof that when applied to clinical samples, any EMT-related signature score, regardless of how well refined it is from preclinical models or scRNA-seq data, becomes a definitive measurement of stromal content rather than epithelial to mesenchymal transition.

### Multi-regional biopsy assessment

We next wished to test the potential clinical implications of these findings, in terms of patient misclassification, using the biopsy of surgical specimens (BOSS; ref. 32) cohort of *n* = 7 primary colon tumor resections, where each patient tumor has bulk transcriptional profiles derived from up to *n* = 5 multi-regional biopsies (Fig. 5E). Application of ssGSEA for the Hallmarks revealed some signature- and patient-specific variations indicative of stromal-derived intratumoral heterogeneity. When assessed individually, all *n* = 5 biopsy samples derived from patient BOSS01 display low expression of all *n* = 17 Hallmarks and *n* = 42 TFs we have previously associated with stroma, in line with this patient having a largely uniform epithelial-rich tumor (Fig. 5F and G). However, the remaining patient samples, particularly from patient BOSS11, BOSS13, and BOSS17, all displayed large variation in gene expression between their patient-matched biopsies for each of the stromal-associated *n* = 17 Hallmark signatures and *n* = 48 TFs (Fig. 5F and G), suggesting that these tumors in particular displayed intratumoral heterogeneity in TSP. To test if the source of this intratumoral heterogeneity in Hallmark scores was due to variation in DS% content across biopsies, we assessed the individual ssGSEA signature scores correlated with the ESTIMATE stromal score which we previously confirmed as an accurate surrogate of DS% (Fig. 4B). Remarkably, these analyses revealed the extent to which stromal content can accurately predict transcriptional signature scores regardless of the patient-of-origin. This was particularly evident for the signatures we have identified to be confounded by stromal content in our LCM, FACS, and bulk tumor datasets, exemplified by positive correlation of EPITHELIAL MESENCHYMAL TRANSITION (Spearman rho = 0.96, *P* = 1.7e-08), KRAS SIGNALING UP (Spearman rho = 0.87, *P* = 7.9e-07), alongside negative correlation with the MYC TARGETS V2 (Spearman rho = -0.63, *P* = 0.00037) signature (Fig. 5H).

### Spatial transcriptomics confirms the confounding effects of the stroma

**Figure 6.**
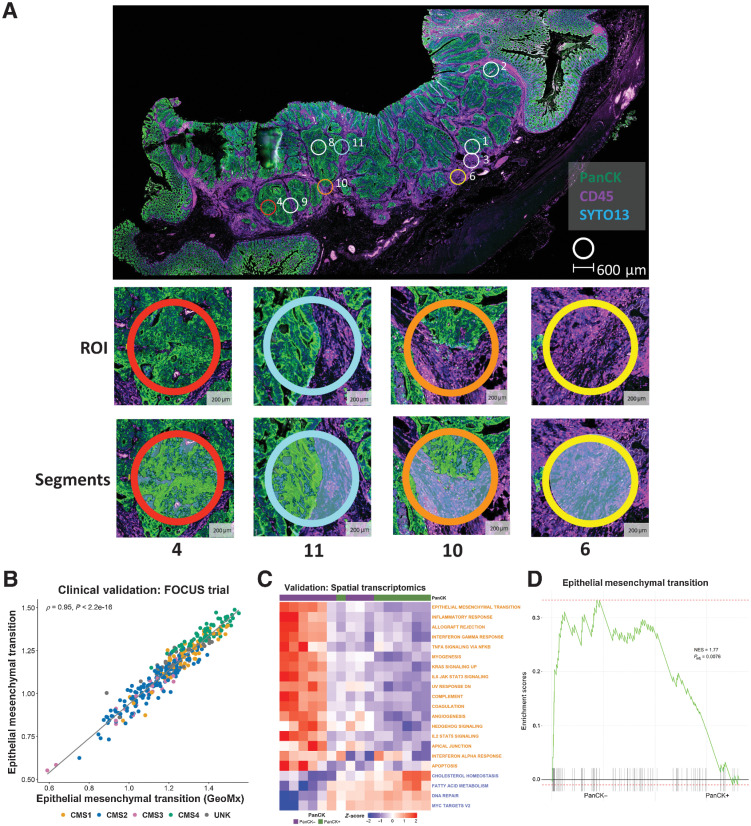
Spatial transcriptomic confirms the confounding effects of the stroma. **A,** Whole slide image of colon cancer case selected for spatial transcriptomic analysis. The tissue was stained with PanCK and CD45 with PanCK^+^ regions (green) identifying epithelium and CD45^+^ regions (purple) identifying immune components. Small circles indicate the ROIs selected for spatial transcriptomic analysis; ROI 4: high epithelial content, ROI 11: mixed epithelial content, ROI 10 demonstrates a ROI with low epithelial content, ROI 6: no epithelial content. **B,** Scatterplot showing the correlation between ssGSEA scores for the full Hallmark Epithelial Mesenchymal Transition gene set (*n* = 200 genes) and the corresponding reduced GeoMx Epithelial Mesenchymal Transition gene set (*n* = 81 genes) in the FOCUS clinical trial cohort (Spearman rho = 0.95). Samples colored by CMS calls (CMS1: *n* = 62; CMS2: *n* = 155; CMS3: *n* = 29; CMS4: *n* = 66; UNK: *n* = 44). **C,** Heatmap of ssGSEA scores for the Hallmark gene sets for the PanCK^+^ (epithelium; *n* = 8) and PanCK^−^ (stroma; *n* = 11) areas within the regions of interest. Only the Hallmark gene sets identified as significantly and concordantly enriched in stroma or epithelium in both the LCM discovery and LCM validation cohorts are shown (the GeoMx versions of these Hallmark gene sets were used). **D,** GSEA comparing PanCK^−^ areas (stroma; *n* = 11) to PanCK^+^ areas (epithelium; *n* = 8) for the Hallmark Epithelial Mesenchymal Transition gene set (GeoMx version). CMS, consensus molecular subtypes; LCM, laser capture microdissection; NES, normalized enrichment scale; ROI, region of interest.

In this study, we have shown the potential for TSP to confound transcriptional signature scores, which in turn can result in misinterpretation of their meaning. Furthermore, analysis in the BOSS cohort also reveal the potential clinical implications of intratumoral stromal heterogeneity, which could result in patient misclassification, or indeed multiple conflicting classifications, when using GESs. To directly assess if spatial transcriptomics (ST) can alleviate some of the confounding variations in transcriptional signaling and inaccurate interpretation of findings when using bulk data, we profiled *n* = 11 regions of a colon tumor sample using the GeoMx ST platform (Fig. 6A). While the GeoMx Cancer Transcriptome Atlas gene panel employed was more limited (*n* = 1,825 core genes in total) when compared with the profiling in our other cohorts; we demonstrated that the reduced total number of genes still represent excellent surrogates for the whole transcriptome by assessing ssGSEA scores of the full signatures alongside the reduced genes available in the ST data. Using data from the FOCUS cohort, we observed excellent concordance in ssGSEA scores of the full MSigDB Hallmark EMT signature (ρ = 0.95; *n* = 200 genes) and MYC Targets V2 signature (ρ = 0.75; *n* = 58 genes), when assessed using the corresponding reduced signatures that were present on the GeoMx panel (*n* = 81 genes and *n* = 8 genes respectively; Fig. 6B; Supplementary Fig. S4A). The ST platform provided the option to stratify our regions of interest into epithelium and stroma, using cytokeratin (PanCK) staining. Using the ST data, we next performed ssGSEA using the Hallmarks we have previously shown to be most confounded by stroma, which again revealed the same general pattern across the *n* = 17 stromal-associated and *n* = 4 epithelial-associated signatures (Fig. 6C). These findings were further confirmed when ST data from across the entire slide was pooled into two groups for pair-wise GSEA, PanCK^−^, and PanCK^+^ (Supplementary Fig. S4B), which again revealed a significant enrichment for the EMT Hallmark signaling cascade in the stromal (PanCK^−^) regions (Fig. 6D). While bulk tumor datasets will remain an essential tool for statistical association studies, these data clearly highlight the need for the compartment and/or lineage-specific stratification, as afforded by ST, to ensure accurate biological interpretation of GESs.

## Discussion

**Figure 7.**
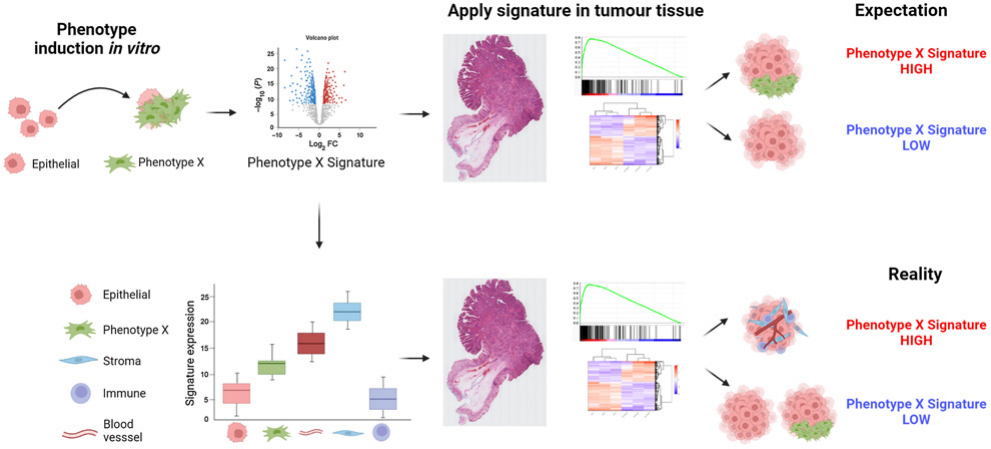
Summary diagram. Precise gene expression signatures have been developed to accurately reflect phenotypic changes in epithelial-based models, when assessed under tightly controlled *in vitro* modeling conditions. When these signatures are used to stratify bulk tumor data, there is an expectation that the same signatures can be used to stratify tumors based on the same distinct phenotypes (top). However, if the genes that make up these signatures are expressed at relatively higher levels in nonepithelial lineages, the signatures can become confounded by even small variations in stromal components, stratifying tumors based on stromal content rather than the phenotype they were developed to represent (bottom).

In this study, we provide a comprehensive characterization of the tumor transcriptome, stratified primarily into epithelium and stroma using LCM and ST, alongside a more granular assessment of individual lineages using FACS and scRNA-seq analysis. These analyses provide insight into the extent of the stroma's contribution to some of the most widely-employed signatures in cancer research, and also the potential for biological misinterpretation of resulting data when extrapolating biology from preclinical models that do not contain a full tumor microenvironment (Fig. 7). Furthermore, we can clearly show the potential clinical implications of these issues in terms of variable patient classification, both through the use of standard annotation and macrodissection to extract and profile RNA from bulk tumor data (as demonstrated in this study from the FOCUS clinical trial) and through the use of multi-regional biopsies from primary resection material. To ensure that all users can benefit from the findings of this study, we have developed a user-friendly and freely-available resource, ConfoundR, which enables assessment of individual genes, pathways, and bespoke signatures across a number of colorectal cancer, breast cancer, TNBC, PDAC, ovarian cancer, and prostate cancer datasets.

Preclinical models, particularly epithelial-rich systems where near-complete control over lineage purity and environmental conditions can be achieved and reproduced, represent ideal systems to develop transcriptional signatures that correlate with phenotypes of interest. We and others have previously highlighted discrepancies between the nomenclature used for such published signatures (6, 33), when named to reflect the phenotypes they characterize *in vitro*, and the actual biology they can represent when applied to bulk tumor datasets. This is primarily due to the fact that while lineage purity is fixed in such preclinical models, a tumor mass is composed of a milieu of lineages (15), the proportions of which are most often unknown at the time of processing for bulk transcriptomic profiling. This becomes particularly problematic for signatures and biomarkers that are developed to characterize mesenchymal traits, as although they will track with precise epithelial biology in *in vitro* systems, when applied to tumor data they are more likely to become highly-accurate tumor stromal percentage estimates, rather than measures of subtle epithelial transitions (7, 8). Our study addresses the importance of also ensuring that the transcriptional signatures faithfully represent the same biology during forward and reverse translation studies, and are not undermined by changes in conditions between clinical and preclinical settings. As such, the most faithful alignment of biological traits between models and clinical samples should be based on deeper phenotyping assessments, that incorporate histology and lineage-specific assessments in addition to transcriptional signatures.

Data presented here gives researchers the opportunity to assess the extent of the stroma's influences on specific genes/signatures of interest to them. The stromal influence on the general transcriptome in bulk tumor data has been demonstrated by Isella and colleagues (3, 4) who identified a set of 4,434 genes present in cancer datasets but exclusively expressed by the tumor stroma. While the nomenclature used in each of the signatures tested may lead researchers to conclude that the biology of that pathway is elevated, data presented here clearly demonstrates that when these otherwise mechanistic-driven signatures are composed of genes expressed at higher levels in stromal cells, the signatures themselves become surrogate markers, to different extents, of the TSP within a bulk tumor sample. Given the extent to which the stroma appears to confound biological interpretation of the thousands of signatures and TFs we have assessed in this study, there may be a significant body of research published that has inadvertently derived conclusions based on inaccurate interpretations of results.

Data presented here do not challenge the use or value of using signatures to interpret data from bulk tumors, but present unambiguous intelligence around the caution that should be applied when interpreting what these signature scores can reflect, despite what the signature name suggests. An inaccurate biological conclusion in itself will not have major consequences for prognostic/predictive signatures in terms of their statistical correlation to identify tumors that are most likely to relapse/respond. However, an issue arises when signatures are being used to describe precise mechanisms across sample types, or when inaccurate biological interpretations of such results are being used as the basis for ongoing clinical therapeutic developments, which themselves are also potentially being tested in models that bear little relevance to the patient tumor samples they are derived from. This is particularly important, as most users are reliant on utilizing existing molecular datasets, where there is no control over the initial profiling steps, or indeed representative histological images that precisely align to the tumor region used for nucleic acid extraction. As such, while the signatures presented here represent large collections of experimentally-validated genes associated with specific phenotypes or biological cascades, our findings support the conclusion that unless users adjust their interpretation based on the extent to which genes/signatures can be influenced by TSP, there is potential for widespread misconceptions when interpreting the meaning underlying transcriptional signatures in tumor studies, given the discordance between their development and application.

Genes can have many functions, and in some cases the genes that strongly demark a specific phenotype in a preclinical model system can also be expressed and perform entirely different mechanistic signaling roles in stromal lineages that make up a TME. If the magnitude of expression of these genes is low in the stromal/immune lineages, this may cause minimal impact when interpreting the precise nature of the transcriptional signature in bulk tumor data. However, if the genes within these signatures are expressed at higher levels in non-epithelial lineages, they can become strong surrogate markers for levels of TME components, rather than reflecting any of the mechanistic biology that they were designed to identify. It is this potential for misinterpretation that our paper wishes to highlight, thereby enabling researchers to assess the potential for their mechanistic signature of choice to be confounded in bulk tumor data using our ConfoundR resource. While data presented here identifies a potential issue in the interpretation of GESs, there is no singular solution given that each gene, and near infinite combination of genes that can be generated to make up signatures, will be influenced to different extents in every individual lineage present in every individual tumor. However, the accompanying ConfoundR resource will allow researchers to make a more informed interpretation of the true biology underlying the signature in these tumor samples, and to adjust their analyses based on the extent to which the TME can influence gene expression of their GES of interest when compared with controlled preclinical models.

Although the use of scRNA-seq analysis can provide high quality lineage-specific transcriptional data, this comes at the expense of spatial information (34). Conversely, ST can regionalize transcriptional signaling but lose the single-cell resolution (35). While both approaches, individually or in combination (36, 37), have revolutionized the field of transcriptional profiling, the use of bulk transcriptomics datasets available in publicly-accessible databases like TCGA and GEO, remain the mainstay for alignment of transcriptional signatures to clinical outcome data for prognostic assessment and mechanistic/biological interpretation. It is likely that with reducing costs and expansion of technologies, the generation of tumor-matched scRNA-seq, spatial and bulk cohorts in both clinical samples and preclinical models will become more routine in future, and at some point may supersede that of existing bulk data. However, as this is unlikely to be in the immediate future, the findings of this study and the unique ConfoundR application we have made publicly-available provide every user the opportunity to assess their gene signatures for themselves, enabling them to adjust their interpretations, if required, of the meaning of their data based on information about the lineage-of-origin of their biomarker or signature when applied to samples with mixed histology. As such, the ConfoundR tool represents an important resource to ensure that translational researchers can more accurately interpret the information underpinning the transcriptional biomarker(s) used to stratify patient samples and inform cancer care.

## Supplementary Material

Supplementary Figure

Supplementary Figure

Supplementary Figure

Supplementary Figure

Supplementary Data

Supplementary Table

## References

[1] Goossens N , Nakagawa S, Sun X, Hoshida Y. Cancer biomarker discovery and validation. Transl Cancer Res 2015;4:256–69. 26213686 10.3978/j.issn.2218-676X.2015.06.04 PMC4511498

[2] Cerami E , Gao J, Dogrusoz U, Gross BE, Sumer SO, Aksoy BA, . The cBio cancer genomics portal: an open platform for exploring multidimensional cancer genomics data. Cancer Discov 2012;2:401–4. 22588877 10.1158/2159-8290.CD-12-0095 PMC3956037

[3] Isella C , Terrasi A, Bellomo SE, Petti C, Galatola G, Muratore A, . Stromal contribution to the colorectal cancer transcriptome. Nat Genet 2015;47:312–9. 25706627 10.1038/ng.3224

[4] Isella C , Brundu F, Bellomo SE, Galimi F, Zanella E, Porporato R, . Selective analysis of cancer-cell intrinsic transcriptional traits defines novel clinically relevant subtypes of colorectal cancer. Nat Commun 2017;8:15107. 28561063 10.1038/ncomms15107 PMC5499209

[5] Dunne PD , Alderdice M, O'Reilly PG, Roddy AC, McCorry AMB, Richman S, . Cancer-cell intrinsic gene expression signatures overcome intratumoural heterogeneity bias in colorectal cancer patient classification. Nat Commun 2017;8:15657. 28561046 10.1038/ncomms15657 PMC5460026

[6] Dunne PD , McArt DG, Bradley CA, O'Reilly PG, Barrett HL, Cummins R, . Challenging the cancer molecular stratification dogma: intratumoral heterogeneity undermines consensus molecular subtypes and potential diagnostic value in colorectal cancer. Clin Cancer Res 2016;22:4095–104. 27151745 10.1158/1078-0432.CCR-16-0032

[7] McCorry AM , Loughrey MB, Longley DB, Lawler M, Dunne PD. Epithelial-to-mesenchymal transition signature assessment in colorectal cancer quantifies tumour stromal content rather than true transition. J Pathol 2018;246:422–6. 30105762 10.1002/path.5155 PMC6282832

[8] Loughrey MB , Fisher NC, McCooey AJ, Dunne PD. Comment on "Identification of EMT-related high-risk stage II colorectal cancer and characterisation of metastasis-related genes. Br J Cancer 2021;124:1175–6. 33311590 10.1038/s41416-020-01213-9 PMC7961054

[9] Cancer models for reverse and forward translation. Nat Cancer 2022;3:135. 35228748 10.1038/s43018-022-00346-5

[10] Gavert N , Zwang Y, Weiser R, Greenberg O, Halperin S, Jacobi O, . Ex vivo organotypic cultures for synergistic therapy prioritization identify patient-specific responses to combined MEK and Src inhibition in colorectal cancer. Nat Cancer 2022;3:219–31. 35145327 10.1038/s43018-021-00325-2

[11] Guillen KP , Fujita M, Butterfield AJ, Scherer SD, Bailey MH, Chu Z, . A human breast cancer-derived xenograft and organoid platform for drug discovery and precision oncology. Nat Cancer 2022;3:232–50. 35221336 10.1038/s43018-022-00337-6 PMC8882468

[12] Barretina J , Caponigro G, Stransky N, Venkatesan K, Margolin AA, Kim S, . The cancer cell line encyclopedia enables predictive modelling of anticancer drug sensitivity. Nature 2012;483:603–7. 22460905 10.1038/nature11003 PMC3320027

[13] Day CP , Merlino G, Van Dyke T. Preclinical mouse cancer models: a maze of opportunities and challenges. Cell 2015;163:39–53. 26406370 10.1016/j.cell.2015.08.068 PMC4583714

[14] Gengenbacher N , Singhal M, Augustin HG. Preclinical mouse solid tumour models: status quo, challenges and perspectives. Nat Rev Cancer 2017;17:751–65. 29077691 10.1038/nrc.2017.92

[15] Baghban R , Roshangar L, Jahanban-Esfahlan R, Seidi K, Ebrahimi-Kalan A, Jaymand M, . Tumor microenvironment complexity and therapeutic implications at a glance. Cell Commun Signal 2020;18:59. 32264958 10.1186/s12964-020-0530-4 PMC7140346

[16] Roseweir AK , Park JH, Hoorn St, Powell AG, Aherne S, Roxburgh CS, . Histological phenotypic subtypes predict recurrence risk and response to adjuvant chemotherapy in patients with stage III colorectal cancer. J Pathol Clin Res 2020;6:283–96. 32401426 10.1002/cjp2.171 PMC7578335

[17] Hynes SO , Coleman HG, Kelly PJ, Irwin S, O'Neill RF, Gray RT, . Back to the future: routine morphological assessment of the tumour microenvironment is prognostic in stage II/III colon cancer in a large population-based study. Histopathology 2017;71:12–26. 28165633 10.1111/his.13181

[18] Calon A , Lonardo E, Berenguer-Llergo A, Espinet E, Hernando-Momblona X, Iglesias M, . Stromal gene expression defines poor-prognosis subtypes in colorectal cancer. Nat Genet 2015;47:320–9. 25706628 10.1038/ng.3225

[19] Guinney J , Dienstmann R, Wang X, de Reynies A, Schlicker A, Soneson C, . The consensus molecular subtypes of colorectal cancer. Nat Med 2015;21:1350–6. 26457759 10.1038/nm.3967 PMC4636487

[20] Mukaka MM . Statistics corner: A guide to appropriate use of correlation coefficient in medical research. Malawi Med J 2012;24:69–71. 23638278 PMC3576830

[21] Vargas AJ , Harris CC. Biomarker development in the precision medicine era: lung cancer as a case study. Nat Rev Cancer 2016;16:525–37. 27388699 10.1038/nrc.2016.56 PMC6662593

[22] Simonyan K , Zisserman A. Very deep convolutional networks for large-scale image recognition. arXiv 2014:1409.556.

[23] Domingo E , Chatzipli A, Richman S, Blake A, Hardy C, Whalley C, . Abstract 4446: Assessment of tissue composition with digital pathology in colorectal cancer. Cancer Res 2019;79 Suppl 13:4446-.

[24] Becht E , Giraldo NA, Lacroix L, Buttard B, Elarouci N, Petitprez F, . Estimating the population abundance of tissue-infiltrating immune and stromal cell populations using gene expression. Genome Biol 2016;17:218–016–1070–5. 27765066 10.1186/s13059-016-1070-5 PMC5073889

[25] Eide PW , Bruun J, Lothe RA, Sveen A. CMScaller: an R package for consensus molecular subtyping of colorectal cancer pre-clinical models. Sci Rep 2017;7:16618. 29192179 10.1038/s41598-017-16747-x PMC5709354

[26] Subramanian A , Tamayo P, Mootha VK, Mukherjee S, Ebert BL, Gillette MA, . Gene set enrichment analysis: a knowledge-based approach for interpreting genome-wide expression profiles. Proc Nat Acad Sci USA 2005;102:15545–50. 16199517 10.1073/pnas.0506580102 PMC1239896

[27] Liberzon A , Birger C, Thorvaldsdottir H, Ghandi M, Mesirov JP, Tamayo P. The Molecular Signatures Database (MSigDB) hallmark gene set collection. Cell Syst 2015;1:417–25. 26771021 10.1016/j.cels.2015.12.004 PMC4707969

[28] Garcia-Alonso L , Iorio F, Matchan A, Fonseca N, Jaaks P, Peat G, . Transcription factor activities enhance markers of drug sensitivity in cancer. Cancer Res 2018;78:769–80. 29229604 10.1158/0008-5472.CAN-17-1679 PMC6522379

[29] Malla SB , Fisher DJ, Domingo E, Blake A, Hassanieh S, Redmond KL, . In-depth clinical and biological exploration of DNA damage immune response as a biomarker for oxaliplatin use in colorectal cancer. Clin Cancer Res 2021;27:288–300. 33028592 10.1158/1078-0432.CCR-20-3237 PMC7614625

[30] Yoshihara K , Shahmoradgoli M, Martinez E, Vegesna R, Kim H, Torres-Garcia W, . Inferring tumour purity and stromal and immune cell admixture from expression data. Nat Commun 2013;4:2612. 24113773 10.1038/ncomms3612 PMC3826632

[31] Özdemir Berna C , Pentcheva-Hoang T, Carstens Julienne L, Zheng X, Wu C-C, Simpson Tyler R, . Depletion of carcinoma-associated fibroblasts and fibrosis induces immunosuppression and accelerates pancreas cancer with reduced survival. Cancer Cell 2014;25:719–34. 24856586 10.1016/j.ccr.2014.04.005 PMC4180632

[32] Ubink I , Elias SG, Moelans CB, Lacle MM, van Grevenstein WMU, van Diest PJ, . A novel diagnostic tool for selecting patients with mesenchymal-type colon cancer reveals intratumor subtype heterogeneity. J Natl Cancer Inst 2017;109. 10.1093/jnci/djw303 28376187

[33] Morris JS , Kopetz S. Tumor microenvironment in gene signatures: critical biology or confounding noise? Clin Cancer Res 2016;22:3989–91. 27334836 10.1158/1078-0432.CCR-16-1044 PMC4987158

[34] Lim B , Lin Y, Navin N. Advancing cancer research and medicine with single-cell genomics. Cancer Cell 2020;37:456–70. 32289270 10.1016/j.ccell.2020.03.008 PMC7899145

[35] Marx V . Method of the Year: spatially resolved transcriptomics. Nat Methods 2021;18:9–14. 33408395 10.1038/s41592-020-01033-y

[36] Andersson A , Bergenstrahle J, Asp M, Bergenstrahle L, Jurek A, Fernandez Navarro J, . Single-cell and spatial transcriptomics enables probabilistic inference of cell type topography. Commun Biol 2020;3:565. 33037292 10.1038/s42003-020-01247-y PMC7547664

[37] Longo SK , Guo MG, Ji AL, Khavari PA. Integrating single-cell and spatial transcriptomics to elucidate intercellular tissue dynamics. Nat Rev Genet 2021;22:627–44. 34145435 10.1038/s41576-021-00370-8 PMC9888017

