# Supplementary material for: Biological Misinterpretation of Transcriptional Signatures in Tumor Samples Can Unknowingly Undermine Mechanistic Understanding and Faithful Alignment with Preclinical Data
Source: Clin Cancer Res. 2022 Jul 6;28(18):4056–69. doi: 10.1158/1078-0432.CCR-22-1102 (PMC9475248; doi:10.1158/1078-0432.CCR-22-1102)
Supplement: Supplementary Figure [file ccr-22-1102_supplementary_figure_s1_supps1.pdf]

Supplementary Figure 1

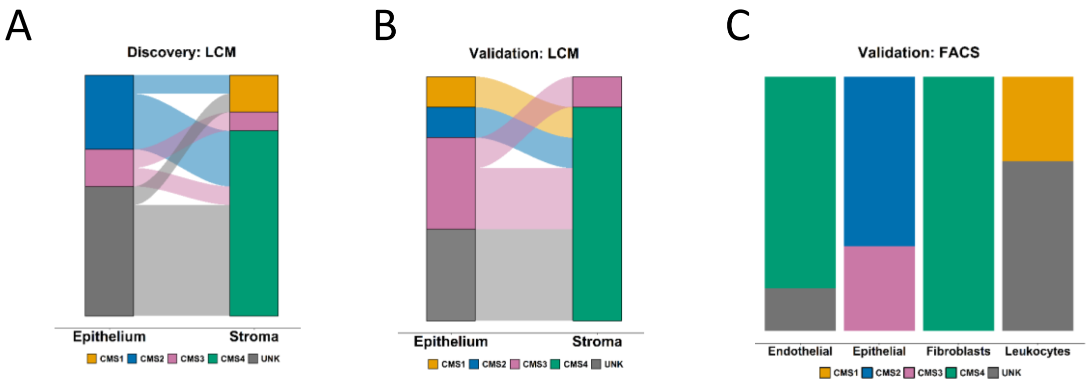

Supplementary Figure 1

A CMS calls (using CMScaller) for the matched epithelium and stroma samples in the LCM discovery cohort. **B** CMS calls (using CMScaller) for the matched epithelium and stroma samples in the LCM validation cohort. **C** CMS calls (using CMScaller) for the four lineages in the FACS validation cohort.
