# Supplementary material for: Biological Misinterpretation of Transcriptional Signatures in Tumor Samples Can Unknowingly Undermine Mechanistic Understanding and Faithful Alignment with Preclinical Data
Source: Clin Cancer Res. 2022 Jul 6;28(18):4056–69. doi: 10.1158/1078-0432.CCR-22-1102 (PMC9475248; doi:10.1158/1078-0432.CCR-22-1102)
Supplement: Supplementary Figure [file ccr-22-1102_supplementary_figure_s3_supps3.pdf]

Supplementary Figure 3

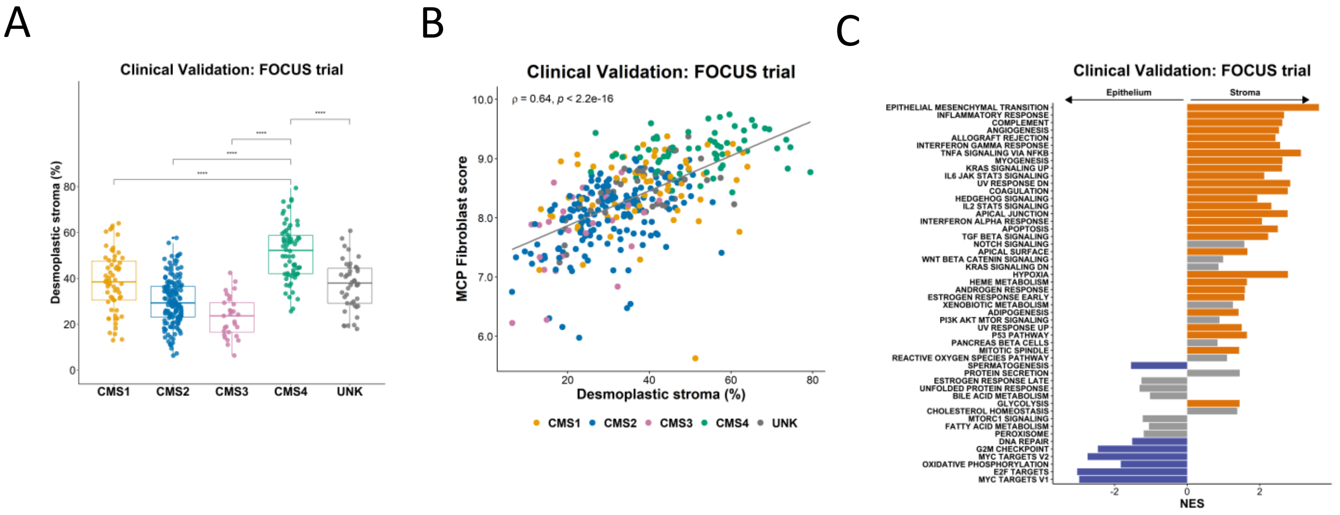

Supplementary Figure 3

A Boxplots showing desmoplastic stroma percentage across Consensus Molecular Subtype (CMS) calls (CMS1: n=62; CMS2: n=155; CMS3: n=29; CMS4: n=66; UNK: n=44) within FOCUS clinical trial cohort (**** p < 0.0001, t test). **B** Scatterplot showing correlation between desmoplastic stroma percentage determined from H&E and Fibroblast score determined by MCP-counter within the FOCUS clinical trial cohort (Spearman's rho = 0.64, p < 2.2e-16) coloured by CMS calls (CMS1: n=62; CMS2: n=155; CMS3: n=29; CMS4: n=66; UNK: n=44). **C** GSEA of Hallmark gene sets comparing samples with above median desmoplastic stroma percentage to samples with below median desmoplastic stroma percentage. Orange bars indicate gene sets significantly enriched in samples with above median desmoplastic stroma percentage while blue bars indicate gene sets significantly enriched in samples with below median desmoplastic stroma percentage (adjusted p-value < 0.02). Only the gene sets significantly and concordantly enriched in stroma or epithelium in both the LCM discovery and LCM validation cohorts are shown and gene sets are arranged in same order as in the LCM discovery cohort (Supplementary Figure 2A).
