# Supplementary material for: Biological Misinterpretation of Transcriptional Signatures in Tumor Samples Can Unknowingly Undermine Mechanistic Understanding and Faithful Alignment with Preclinical Data
Source: Clin Cancer Res. 2022 Jul 6;28(18):4056–69. doi: 10.1158/1078-0432.CCR-22-1102 (PMC9475248; doi:10.1158/1078-0432.CCR-22-1102)
Supplement: Supplementary Figure [file ccr-22-1102_supplementary_figure_s4_supps4.pdf]

Supplementary Figure 4

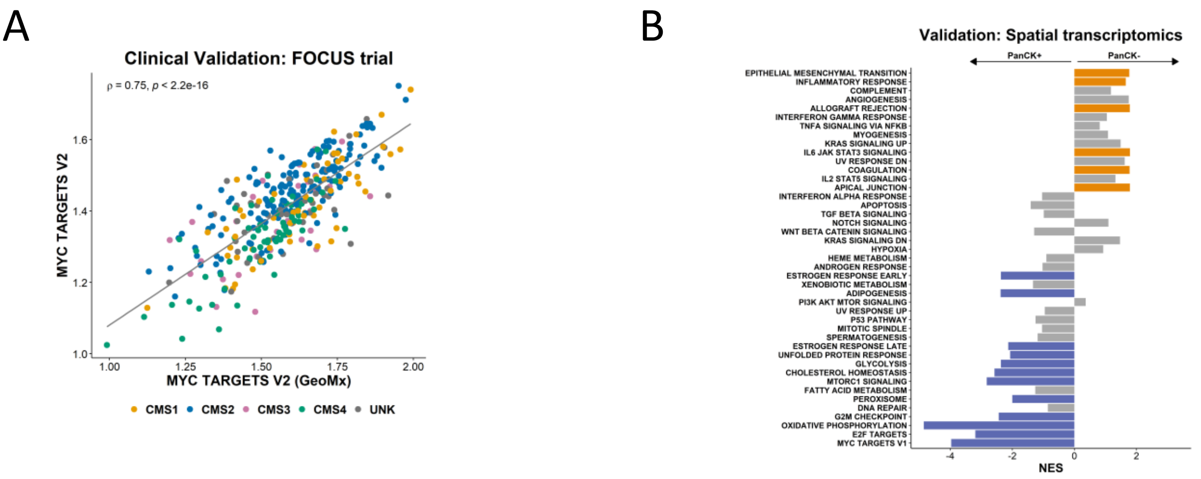

Supplementary Figure 4

A Scatterplot showing the correlation between ssGSEA scores for the full Hallmark MYC Targets V2 gene set and the GeoMx MYC Targets V2 gene set in the FOCUS clinical trial cohort (Spearman's rho = 0.75, $p < 2.2e\text{-}16$). Samples coloured by CMS subtype (CMS1: n=62; CMS2: n=155; CMS3: n=29; CMS4: n=66; UNK: n=44). **B** GSEA comparing PanCK- areas (stroma) to PanCK+ areas (epithelium) for the Hallmark gene sets (GeoMx versions). Orange bars indicate gene sets significantly enriched in PanCK- areas (stroma) (n=11) while blue bars indicate gene sets significantly enriched in PanCK+ areas (epithelium) (n=8) (adjusted p-value < 0.02).
