# Supplementary material for: Biological Misinterpretation of Transcriptional Signatures in Tumor Samples Can Unknowingly Undermine Mechanistic Understanding and Faithful Alignment with Preclinical Data
Source: Clin Cancer Res. 2022 Jul 6;28(18):4056–69. doi: 10.1158/1078-0432.CCR-22-1102 (PMC9475248; doi:10.1158/1078-0432.CCR-22-1102)
Supplement: Supplementary Data [file ccr-22-1102_supplementary_methods_suppsm.docx]

**GeoMx *Digtial Spatial Profiler***

### Nanostring GeoMx Digital Spatial Profiling

To further characterize differences in transcriptomic expression between TME and tumor epithelium, a formalin-fixed paraffin-embedded (FFPE) section of archival resected colon cancer was selected for analysis on the Nanostring GeoMx Digital Spatial Profiler (DSP). This platform enables the characterization of user-selected topographic Regions of Interest (ROI) from immunofluorescently (IF) stained FFPE tissue. The GeoMx instrument achieves RNA profiling *in situ* hybridization by employing DNA oligonucleotide probes designed to bind mRNA targets. From 5′ to 3′, they comprise a 35- to 50-nucleotide target complementary sequence, an ultraviolet (UV) photocleavable linker and a 66-nucleotide indexing oligonucleotide sequence containing a unique molecular identifier (UMI), RNA ID sequence and primer binding sites. Up to 10 RNA detection probes were designed per target mRNA. In summary, the instrument employs UV light to cleave the UV-sensitive probes leading to release of the hydridized barcodes.

#### *Slide preparation including hydridization of tissue with UV-photocleavable probes*

The DSP procedure has previously been described in detail by Merritt et al (1). The 5-µm FFPE tissue section was mounted on positively charged Superfrost glass slide (Thermo Fisher Scientific) and baked for 30 mins at 60 °C. The tissue was dewaxed, hydrated and treated with 1μg/ml Proteinase K (Thermo Fisher Scientific, AM2546) for 15 minutes before undergoing heat-induced epitope retrieval (HIER) on a Leica BOND Autostainer (pH 9.0, ER2 at 100°C) for 20 minutes. The slide was immediately stored in 1X PBS (PBS: Invitrogen, AM9625). Hydridization with a pre-designed Cancer Transcriptome Atlas (CTA) panel of antibodies corresponding to 1,825 genes (Nanostring) was performed according to the manufacturer’s protocol (2). 100μL of the RNA probe mix (CTA panel) was mixed with 800μL of Buffer R (Nanostring) and 100μL of DEPC-treated water. Each tissue was covered with 200μL of hydridization solution and a HybriSlip™ cover (Thermo Fisher Scientific) before overnight incubation in a hydridization oven at 37 °C for at least 16 hours. The slide was then dipped in 2X SSC-T and washed twice with a 1:1 ratio of 100% deionized formamide (Ambion) and 4X SSC (Sigma) at 37°C for 25 minutes each.

The GeoMx DSP is capable of capturing four channels (FITC/525nm, Cy3/568nm, Texas Red/615nm and Cy5/666nm) for the detection of up to four customizable IF morphology markers for each tissue (1). One channel (FITC/525nm) is reserved for the nuclear stain (SYTO13). The slides were blocked with Buffer W (Nanostring) for 30 minutes at RT before incubation with TME RNA Morphology Marker kit (Nanostring) for 1 hour at RT. This consisted of fluorescently conjugated Syto13, Pan-Cytokeratin (PanCK) and CD45 antibodies were used to stain the tissue to identify nuclei, tumor epithelium and the immune components respectively. Slides were then stored at 4°C in SSC before being loaded on the GeoMx DSP instrument for ROI selection and collection.

#### *Region selection and collection*

The whole slide was imaged at 20x magnification using the GeoMx DSP with the integrated software suite then used to select 300-600um diameter ROIs from which the instrument focuses UV light (385nm), to cleave the UV-sensitive probes with the subsequent release of the hydridized barcodes. 11 ROIs corresponding to epithelial tumor center, abundant TME regions and regions representing an interface between tumor and TME and were selected. The DSP software enabled Areas of Interest (AOI) contained in individual ROIs to be defined and selected. Firstly segments containing PanCK+ IF signal were masked for tumor epithelium and extracted, then the complementary inverse segments (PanCK-) was masked and captured corresponding to the TME. Once AOIs were defined, then exposed UV light, the indexing oligonucleotides, were collected with a microcapillary and deposited in a 96-well plate prior to sequencing. The oligonucleotides were dried overnight and subsequently resuspended using 10μl of DEPC-treated water

#### *Library Preparation and NGS Sequencing*

Sequencing libraries were generated by PCR from the photo-released indexing oligos and AOI-specific Illumina adapter sequences, and unique i5 and i7 sample indices were added. Each PCR reaction used 4µl of each collection sample added to the corresponding well of a new 96-well PCR plate containing the GeoMx Seq Code primers (Nanostring) and 1X PCR Master Mix (Nanostring). The PCR plate was incubated in a thermocycler with the program specified by the manufacturer. The PCR products were then centrifuged and pooled (4μl each) into one 1.5 mL Eppendorf to create a library. The library was purified twice using AMPure XP system (Beckman Coulter). The purified library was resuspended in Elution Buffer (10mM Tris-HCl with 0.05% Tween-20, pH 8.0) before undergoing quality check using an Agilent Bioanalyzer.

The purified library underwent Next Generation Sequencing (NGS) using an Illumina NextSeq 550 (Glasgow Polyomics). Recommended sequencing parameters were followed in generation of FASTQ files, dual-indexing, paired-end reads and including a 5% PhiX spike-in. Sequencing depth was determined by total ROI area (µm^2^) multiplied by sequencing depth factor (30 for CTA panel) as per manufacturer’s instructions. H&Es from Focus (N=356) were scanned at high resolution on an Aperio scanner at a total magnification of 20X. Tissue segmentation was run on H&E images by deep convoluted neural net using the HALO platform (Indica Labs). Supervised training had been performed using >1,500 tissue areas from four CRC cohorts.

*Data analysis*

The FASTQ files generated were converted into Digital Count Conversion (DCC) files using the GeoMx NGS pipeline on the Illumina BaseSpace platform. The DCC files were uploaded onto the GeoMx DSP analysis suite (Nanostring), where they underwent quality control, filtering, Q3 normalization and background correction. Data were then downloaded from the GeoMx instrument and loaded in to RStudio (v1.2.1335) using R build version 4.1.1.
